# Psychological Correlates of Self-Reported and Objectively Measured Physical Activity among Chinese Children—Psychological Correlates of PA

**DOI:** 10.3390/ijerph13101006

**Published:** 2016-10-13

**Authors:** Jing-Jing Wang, Tom Baranowski, Patrick W. C. Lau, Tzu-An Chen, Shu-Ge Zhang

**Affiliations:** 1 Department of Physical Education, Faculty of Social Sciences, Hong Kong Baptist University, Hong Kong, China; wangjj@life.hkbu.edu.hk (J.-J.W.); sugar.zhangshuge@gmail.com (S.-G.Z.); 2 Children’s Nutrition Research Center, Department of Pediatrics, Balor College of Medicine, Houston, TX 77030, USA; Thomas.Baranowski@bcm.edu; 3 Center for Translational Injury Research, University of Texas Health Science Center at Houston, Houston, TX 77030, USA; Tzu-An.Chen@uth.tmc.edu

**Keywords:** psychological correlates, accelerometry, self-report, physical activity

## Abstract

This study aimed to explore the associations among psychological correlates and physical activity (PA) in Chinese children and to further examine whether these associations varied by different PA measures. PA self-efficacy, motivation, and preference were reported in 449 8–13-year-old Chinese children (252 males). Moderate- to vigorous- intensity PA (MVPA) was measured by the Physical Activity Questionnaire for Older Children (PAQ-C) and with an ActiGraph GT3X accelerometer. Correlations and hierarchical regressions were performed to explore their associations. The study psychological variables were all positively related to PAQ-C and objective MVPA (*r*: 0.22–0.63). The associations with PAQ-C were all substantially stronger than those with accelerometry. Beyond the explained variance accounted for by demographics and social desirability, the addition of the psychological correlates accounted for 45% of the variance of the PAQ-C score, while only 13% for accelerometry-based MVPA. The associations of specific variables with the PAQ-C score (age, PA self-efficacy, autonomous motivation and preference) were somewhat different from those associated with objective MVPA (PA self-efficacy, autonomous motivation, and negatively associated with female gender). This study demonstrated the importance of self-efficacy and autonomous motivation in association with PA and indicated the difference in level of their associations with different PA measures.

## 1. Introduction

The health benefits of regular physical activity (PA) in children have been well documented, including reduced body fat, enhanced physical fitness and bone health, more favorable cardiovascular risk profiles, and decreased symptoms of anxiety and depression [1]. Current PA guidelines recommend children aged 5–17 years to participate in at least 60 min of moderate-to vigorous-intensity PA (MVPA) on a daily basis [2]. Despite considerable evidence supporting the protective effects of PA, physical inactivity remains widespread [3]. A national survey using accelerometers revealed that Chinese children and youth spent an average of 28.3 min per day in MVPA. Only 9.4% of boys and 1.9% of girls met the recommendation of 60 min/day of MVPA [4]. Similarly, in Hong Kong, only 8.3% of children aged 7–12 years engaged in the recommended PA levels [5].

Interventions that target strong and consistent modifiable correlates of behavior should be more effective in changing behavior [6]. Given the low levels of PA in China, understanding the association between correlates and PA could inform the development of more efficacious interventions. Psychological factors have been extensively identified as an important category in reviews concerning correlates of PA in children and adolescents [7,8]. In the PA domain, self-efficacy beliefs are emphasized as a situation-specific of self-confidence that people have to engage in PA [9,10]. Self-efficacy is positively associated with exercise adherence [11]. A few studies have examined the positive relationship between self-efficacy and PA in Chinese pediatric populations in mainland China [12,13], Taiwan [14,15], and Hong Kong [16]. However, only self-reported PA data were collected in these studies.

Self-determination theory (SDT) [17] is another appealing theoretical framework for understanding how motivational factors may relate to PA and has been widely employed. SDT posits that motivation helps individuals initiate and maintain behavior. SDT offers a motivational sequence ranging from low to high levels of self-regulation underpinning PA, known as amotivation (i.e., lack of intentionality and personal causation), external regulation (i.e., external locus of initiation, e.g., for gaining reward or avoiding punishment), introjected regulation (i.e., involving internalized rules or demands), identified regulation (i.e., realizing the value of behavior and accepting the regulatory process), and intrinsic motivation (i.e., inherent satisfaction in doing the behaviors) [18]. Based on these different motivational regulations, SDT distinguishes the autonomous and controlled motivations. Intrinsic motivation and identified regulation are considered autonomous forms of motivation because they reflect a sense of personal volition and originate from an internal perceived locus of causality. Alternatively, introjected and external regulations are considered controlled motivation to reflect external demands, originating from an external perceived locus of causality [19].

SDT provides valuable insight into how to foster increments in autonomous motivation, indicating that more self-determined forms of motivation lead to optimal functioning and well-being [20]. A review of forty-six studies of the association between the motivations and PA in children and adolescents indicated that autonomous motivation had a moderate positive association with PA (*ρ* = 0.27 to 0.38), whereas controlled forms of motivation had weaker negative associations with PA (*ρ* = −0.11 to −0.21) [21]. Most of these existing studies, however, were derived from western populations. One study among Chinese students found differences in PA levels between high and low self-determined groups [22]. Two PA motivation studies were conducted in mainland China and Taiwan [23,24], but were specifically conducted in physical education contexts. There is a paucity of research on PA motivation among underserved Chinese children in free-living conditions. Considering the obvious western vs. Chinese cultural differences (e.g., heavy homework load and emphatic stress on academic performance among the Chinese), perceived pressure on school work might influence Chinese children’s intentions and decisions to be physically active [25]. Thus, studies of Chinese children’s motivational correlates of daily PA are imperative.

Furthermore, preference for the behavior, a component of behavioral choice theory [26], has been applied to explain PA behavioral choice. Preference for PA was a significant predictor of engagement in PA in a large community-based sample of adults [27]. Clustering of activity preferences was identified in primary school children [28], with higher preference or liking for sedentary behavior negatively associated with time spent in a free-choice situation [29]. However, few studies have assessed the association between children’s PA preference with active behaviors.

**Figure 1:**
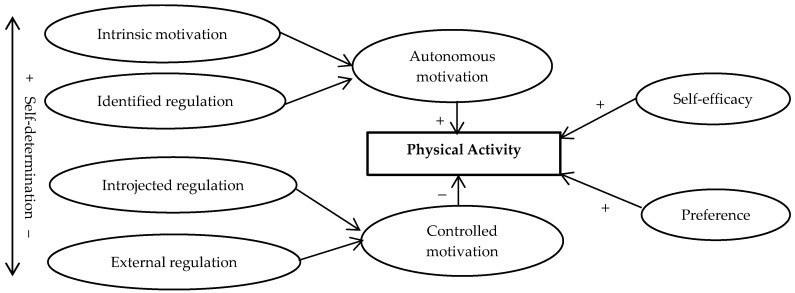
Hypothesis of study psychological correlates and physical activity behaviors.

This study aims to assess the associations among several psychological factors (i.e., self-efficacy, preference, motivation) and PA in Chinese children. We hypothesized that self-efficacy, preference, and autonomous motivation would be positively associated with PA, whereas, controlled motivations would be in negative association with PA (Figure 1). Moreover, given inherent limitations and bias in self-reported measures of PA [30], research is needed using more objective measure, which is paramount to our attempts to better explore the correlates of actual PA behaviors. In the current study, children’s PA was measured with both an accelerometer and self-reported recall questionnaire to assess the possible differences in these relationships.

## 2. Materials and Methods

### 2.1. Participants

Seven Hong Kong primary schools that approved to participate in the study were included. The schools were located in different Hong Kong districts which varied in student social-economic status (SES) (two from high SES, two from medium SES, and three from low SES) according to local statistics [31]. Students with any contraindication to PA, physical disease (heart, lung, liver, kidney, other vital organs, endocrine diseases or drug side effects), or psychological illnesses (depression and anxiety related disorders) that may have prevented them from participating in PA were excluded. The study was volunteer-based. We delivered the study information to students from grades 4 to 6 by physical education teachers. Students who provided written consent forms from each participant and their parents or guardians were included in this study from September 2014 to July 2015. A total of 462 students aged 8 to 13 years (260 males; age mean (SD) = 10.2 (1.1) years; body mass index (BMI) mean (SD) = 18.76 (3.73) kg/m^2^) were recruited. The study obtained ethics approval from the Committee on the Use of Human and Animal Subjects in Teaching and Research of Hong Kong Baptist University (Ref. HASC/Student/13-14/047).

### 2.2. Procedures

The translation of the questionnaires from English to Cantonese Chinese consisted of three separate forward translations by native speakers of the target language, and subsequently back translated by English speakers. Prior to data collection, five Hong Kong Chinese students were invited to test the comprehensibility of the Cantonese questionnaire. Minor wording revisions were made based on their feedback. On the testing day, height and weight were measured and questionnaires were delivered to students. During the completion of the questionnaires, research assistants distributed the accelerometers to students. 

### 2.3. Measures

#### 2.3.1. Height, Weight and Body Mass Index (BMI)

Height was measured to the nearest 0.1 cm using a calibrated FISCO measuring tape (CMS Weighting Equipment Ltd., London, UK) and weight was measured to the nearest 0.1 kg using a Tanita electronic digital scale (Model No. HD305, Tanita Inc., Tokyo, Japan) complying with standard anthropometric methods. BMI (kg/m^2^) was calculated as weight divided by height squared. 

#### 2.3.2. Objective Physical Activity Behaviors

Objective physical activity was measured using ActiGraph GT3X accelerometers (AG: ActiGraph LCC, Fort Walton Beach, FL, USA), which have demonstrated high reliability and validity among children [32]. Students were asked to wear the device positioned on the right hip for 7 consecutive days during waking hours. The accelerometer could only be removed during water-related activities (swimming, showering, and bathing) and while sleeping, and any removal was to be recorded in the PA diary given to the students. The diary was used to improve compliance to wearing the accelerometers. AG is a small, lightweight, and unobtrusive triaxial device that measures acceleration in activity counts and step counts at pre-selected epochs. In the present study, 5-s epochs were used. Activity counts were summed as per minute intervals. For analysis, extreme values (>20,000 counts per min (CPM)) were removed. No less than 8 h of valid wearing time with no more than 20 min of consecutive zeroes were recognized as a valid day. After one-week of wearing, children who could provide a minimum of 3 valid days (including one weekend day) were included in the final analyses [33]. Based on recent recommendations [34], cut-off points developed by Evenson et al. [35] were used to determine the intensity of MVPA (≥2296 CPM) in children. 

#### 2.3.3. Self-Reported Physical Activity Behaviors

Self-reported physical activity was measured using the Physical Activity Questionnaire for Older Children (PAQ-C) validated for use with Chinese children (*α* = 0.78) [36]. PAQ-C includes ten items, nine of which are used to compute the score that measure general MVPA for the day as a whole and for segments during the day (e.g., physical education class, recess, lunchtime, after school, evening, weekends) or day of week using a 5-point Likert scale with higher scores indicating higher PA levels. The last item identifies whether sickness or other events prevented the child from participating in their regular PA, and is not included in the calculation of the activity scores.

#### 2.3.4. Physical Activity Self-Efficacy

Physical activity self-efficacy was assessed using a validated 12-item Physical Activity Self-efficacy scale developed by Jago and colleagues (*α* = 0.92) [37]. Example items included “How sure are you that you can be physically active more than 30 min for one day, even when you have homework?” “How sure are you that you have the ability to do physical activities such as running, dancing, bicycling, or jumping rope?” (1 = I am not sure; 2 = I am sure a little; 3 = I am sure a lot).

#### 2.3.5. Physical Activity Preferences

Physical activity preferences were measured using a validated 28-item Self Administrated Physical Activity Checklist (*α* = 0.85) [38]. Items asked how much children liked the different PA (e.g., bicycling, swimming, dancing, etc.) (1 = I have never done it; 2 = I do not like it; 3 = I like it a little; and 4 = I like it a lot). The scale averaged 28 items to create an overall measure of PA preference. High scores represent a greater preference for PA. 

#### 2.3.6. Physical Activity Motivation

The Motivation for Exercise Questionnaire that consists of 16 items was used to assess an individual’s four types of behavioral self-regulation derived from SDT: three items for intrinsic motivation, five items for identified regulation, three items for introjected regulation, and five items for external regulation [39]. Children scored these items for the reasons why they engage in PA by using a 7-point Likert scale (1 = not at all true; 4 = somewhat true; 7 = very true). Example items are: I am active regularly “Because I enjoy being active” (intrinsic motivation), “Because it is a challenge to accomplish my goal” (identified regulation), “Because I would feel like a failure if I was not active” (introjected regulation), “Because others would be angry at me if I was not active” (external motivation). Standardized coefficient alphas in the current sample were 0.86 for intrinsic, 0.81 for identified, 0.72 for introjected, and 0.73 for external regulation. The score for autonomous motivation (*α* = 0.89) was created by averaging the items in the subscales of intrinsic motivation and identified regulation, whereas the items under introjected and external regulations were averaged to form a score of controlled motivation (*α* = 0.80).

#### 2.3.7. Social Desirability (SocD)

Since previous studies found that social desirability (SocD), the self-report bias of overestimating desirable behaviors and underestimating undesirable ones, influenced self-reports of PA as a source of error [40], SocD was measured and statistically adjusted in the current study. SocD was assessed using a nine-item Lie Scale of Children’s Manifest Anxiety Scale [41] with good internal consistency in the current sample (*α* = 0.89). Sample items included “I tell the truth every single time; I never say things I shouldn’t” (0 = never true of me; 1 = not sure; 2 = sometimes true of me; 3 = always true of me).

### 2.4. Statistical Analysis

Data were entered into Epidata 3.1 and analyzed with SPSS 20.0. Data were screened for outliers and missing values. Descriptive statistics were computed to describe the qualified participants’ characteristics. A multivariate analysis of variance (MANOVA) was employed to investigate gender differences in intrinsic motivation, and identified, introjected and external regulations. Independent samples *t* tests were calculated to examine gender differences for mean values of PA self-efficacy, preference, and behaviors. Bivariate Pearson correlations were computed to explore the relationships among study variables. Partial correlation coefficients would be reported with adjustment for gender and age if PA behavior differed across gender and age groups. Correlation coefficients being lower than 0.35, ranging from 0.36 to 0.67, and higher than 0.68 are considered to represent weak, moderate, and strong correlations, respectively [42]. Hierarchical regression was performed to evaluate the associations of motivation, self-efficacy, and preference on both self-reported and objective MVPA. For each of the two regression analyses, gender, age, BMI and SocD were entered in the first Step, and PA self-efficacy, preference, autonomous and controlled motivations were entered in Step 2. The significance of the *F* ratio accompanying the change in variance (*R*^2^) for each step indicated the significance of the addition of each group of independent variables to the regression equation. If the step was significant, then the standardized coefficients (B), standard error (SE) and *t* values of each independent variable within the regression equation were reported.

## 3. Results

### 3.1. Participants’ Demographic Characteristics

Of 462 participants, 13 were excluded due to missing data or events that prevented them from engaging in regular PA during the previous week and 449 children (56.1% males) were included for final analysis. No extreme scores were identified. Of 449 participants, 365 children (81.3%) with 200 males and 165 females provided valid accelerometer data. There were no differences in demographics or anthropometrics between those that provided objective PA data and those that did not. Children reported a PAQ-C score at 2.71 ± 0.70. Average time spent in MVPA was 43.1 (SD: 12.7) min/day. Boys (45.3 ± 13.3 min/day) were more physically active than girls (40.4 ± 11.5 min/day) based on accelerometer-assessed MVPA (*t* = 3.7, *p* < 0.001). Differences in objective MVPA were also found across age groups (*F* = 2.9, *p* = 0.035) (Table 1).

### 3.2. Preliminary Analyses on Psychological Correlates and PA Behaviors

Descriptive statistics, internal consistency reliability coefficients and bivariate correlations among the psychological correlates and PA behaviors are shown in Table 2. Participants had an average PA self-efficacy score of 2.1 (SD: 0.6), and PA preferences score of 2.9 (SD: 0.4). Children reported the intrinsic motivation, identified, introjected, and external regulations at 5.3 ± 1.7, 5.0 ± 1.6, 3.1 ± 1.7, 2.4 ± 1.3, respectively. Children endorsed autonomous motivations (5.1 ± 1.5) more highly than controlled motivations (2.6 ± 1.3). Boys had higher controlled motivations than girls (introjected regulation: *F* = 5.8, *p* = 0.02; external regulations: *F* = 7.4, *p* < 0.001). No significant gender differences were detected on other psychological correlates.

Moderate correlations were found among self-efficacy, preference, and autonomous motivation (all *r* > 0.50, *p* < 0.01), while relatively low correlations were found between these variables and controlled motivation (*Ps* < 0.01) (Table 2). External regulation underpinning controlled motivation was unrelated to PA preferences (*p* > 0.05). PAQ-C and objective MVPA correlated at *r* = 0.39 (*p* < 0.01). PA self-efficacy, preferences, autonomous and controlled motivation were all positively related to PAQ-C score and objective MVPA with correlation coefficients ranging from 0.22 to 0.63 (*Ps* < 0.01). External motivation was weakly correlated to the PAQ-C score (*r* = 0.20, *p* < 0.01), but unrelated to objective MVPA (*p* > 0.05). The relationships between the psychological correlates and PAQ-C were all substantially stronger than those measured by accelerometer.

### 3.3. Associations of Age, Sex, BMI, Social Desirability and Psychological Correlates with PA Behaviors in the Hierarchical Regression Models

Multiple step-wise regression analyses examined whether the psychological constructs correlated with PA levels. Only children providing both valid PAQ-C and accelerometer data (*n* = 365) were included in the regression procedures. As shown in Table 3, the demographic characteristics and SocD accounted for only 1% of the variance in PAQ-C, while the addition of the psychological correlates accounted for an additional 45% of the variance. In the hierarchical regression model, PAQ-C score was positively associated with age, PA self-efficacy, preferences and autonomous motivation. In the regression model of MVPA assessed by accelerometer, adding the psychological correlates to the regression model only increased explained variance by 13%. Objective MVPA was positively associated with PA self-efficacy and autonomous motivation, and negatively related with gender (indicating that males were more active).

## 4. Discussion

This study is among the first to investigate the importance of psychological correlates in understanding PA of Chinese children using both accelerometry and self-reported estimates, and to demonstrate that the associations varied depending on the different PA measures. The findings of correlation analyses showed that PA self-efficacy, preference, autonomous and controlled motivations were all positively related to PAQ-C score and objective MVPA. The correlations with PAQ-C were all substantially stronger than those with accelerometry. In a hierarchical regression model, age, PA self-efficacy, preferences, and autonomous motivation were positively associated with self-reported PA after controlling for gender, BMI, and SocD. However, age and PA preference did not contribute significantly in the hierarchical model predicting objectively assessed MVPA which was positively associated with PA self-efficacy and autonomous motivation, and negatively associated with female gender.

Self-efficacy has been one of the most important correlates of youth PA [43]. The available research on gender differences in self-efficacy for PA in children and adolescents is somewhat inconsistent among Chinese children. Among Taiwanese adolescents, girls reported lower PA self-efficacy than boys [15], whereas, no gender difference was detected in this study or by another study among Hong Kong Chinese children [16]. In the current study, PAQ-C and objective MVPA were both positively associated with self-efficacy in the hierarchical regression analyses. Previous studies have indicated that time spent in PA was predicted by self-efficacy in Chinese children [13]. Positive association between self-efficacy and PA was also found in Hong Kong children [16], Taiwanese adolescents [14], and South Korean children [44]. Similar to findings among Caucasian children [45], self-efficacy appears to be an important correlate of PA among Asian cultures.

Higher autonomous motivation (i.e., identified and intrinsic) should be a more consistent predictor of reported behavior than higher externally oriented motivations (i.e., controlled motivations consisting of extrinsic and introjected relegations) [18]. Participants in the current study reported higher levels of autonomous motivation and lower levels of controlled motivation. Congruent with previous studies using SDT [46,47], autonomous motivation was positively associated with greater PA for both PAQ-C and objectively-assessed MVPA. This finding suggests that children’s PA levels increase as their belief that PA is inherently enjoyable and pleasurable and the values they place on PA increases. Theoretically, controlled regulation should be negatively associated with PA [20]. The empirical study of Owen et al. [21] demonstrated the negative association between the controlled regulation and exercise behavior. However, departed from the hypothesis, positive association between controlled motivation and PA was observed in the current study. Introjected regulation, underpinning controlled motivation, was positively related to both self-reported and objectively assessed PA, which were also found in the previous studies [46,48]. Introjected regulation is motivation from an internalized, pressuring voice or demand. The positive association between introjected regulation and PA might reflect children’s concern for their physique, e.g., the motivation to engage in PA partly to satisfy self-needs and pressure to have a desired body shape and physical appearance [49]. Additionally, introjected regulation appears to have been associated with PA in the short-term, but not the longer term [50,51]. Due to the limitation of cross-sectional design in this study, the potentially long-term unassociation between introjected regulation and PA cannot be assessed; this implies the need for a persistent emphasis on identifying the value and motivating the enjoyment of the behavior.

This is one of only a few studies that investigated PA preference, which may be key in developing effective PA-promoting and weight-control strategies for children [52]. Preferences for moderate PA accounted for 2.9% of the variance in moderate PA and 16.3% of the variance in vigorous PA among adults [27]. In the current study, hierarchical regression revealed that PA preference was a positive correlate of PAQ-C, but not for objectively assessed MVPA. This discrepancy calls for more research on the association between PA preferences and PA level among Chinese children. PA preference was generally measured with a PA checklist [52,53]. The findings of this study also indicated that regardless of how people perceived they prefer the activities, they may not behave in accordance with their preferences, which may be due to several impending factors, for example, unforeseen barriers, unavailable activity and environmental influences. Knowledge of the types of activities in which children would prefer to participate is of value in planning communication campaigns. The further explicit association between PA preference and PA would be useful to a variety of stakeholders to develop the intervention programs to understand how certain features of PA programs (e.g., intensity and type of PA) can be better tailored to meet the PA interests of Chinese children.

This study examined PA all day, including during school time and after school times, and leisure time PA rather than PA in physical education or other specific settings. Children in this study reported a slightly higher MVPA level (43.1 ± 12.7 min) than Chinese children in another study (28.3 ± 17.7 min) due to the adoption of different cut-points for MVPA (≥2296 CPM in the current study vs. ≥2800 CPM in the other study) [4]. Considering the PA level assessed by accelerometer was sensitive to the cutoff points defining activity intensity, the results from studies using different cutoff points would therefore not be comparable. However, for the PA behaviors across gender groups, consistent with previous literature, this study demonstrated boys engaged in significantly more objective MVPA than girls [54]. In the current study, age was significantly associated with PAQ-C score, but not with objectively assessed PA. In a national sample of American children in grades 4 through 12, a regression analysis of correlates of PA explained more variance in PA in the oldest than the youngest groups [55], suggesting reliability and validity of self-reports likely increase with age [56]. 

A notable strength of the current study was the use of both accelerometer and self-reported measures of PA. Although SocD bias was measured and controlled for in the current study, it was associated with neither self-reported nor objectively assessed PA. The correlation coefficients between psychological correlates with PAQ-C were substantially higher than those with objective MVPA. The psychological correlates accounted for 45% of the variance of the PAQ-C score beyond that explained by demographics and SocD, but increased explained variance by only 13% of objectively assessed MVPA. Previous studies demonstrated similar discrepancies in the contribution of determinants with adolescents’ PA using the subjective and objective PA measures [57,58]. The difference in level of predictiveness is likely due to self-reported error variance common to the PAQ-C and psychological correlates, but not common to accelerometry. Shared method variance may lead to overestimation of the association [59].

Limitations in the present study should be warranted. Firstly, associations investigated were cross-sectional in nature, which precludes casual inferences. Secondly, participants were volunteers, which may have resulted in a “self-selection” bias and thereby limit the generalization. Lastly, although participants’ age, BMI, gender, and SocD were statistically controlled in the analyses, other socio-demographics such as parental education level, household income, peer influences, and parental support may be potential confounders.

## 5. Conclusions

This study was an important step in understanding the strength of the associations between psychological correlates and PA behavior in Chinese children. Higher levels of self-efficacy and autonomous motivation were positively related to PA level. Moreover, the explainable variance of correlates substantially differed by PA measures, suggesting the more research are recommended with an objective measure. In the future, longitudinal research with objective measures is needed to identify the prediction of these two psychological factors in Chinese children. Furthermore, the additional socio-psychological correlates need to be explored to enhance the predictiveness of PA.

## Figures and Tables

**Table 1:** Physical Activity Behaviors among Different Gender and Age Groups.

	PAQ-C Score			Objective MVPA (min/day)		
	n	Mean ± SD	Statistics *(p)*	n	Mean ± SD	Statistics *(p)*
Overall	449	2.71 ± 0.70		365	43.09 ± 12.74	
Gender			1.73 (0.084)			3.66 (<0.001)
Males	252	2.76 ± 0.71		200	45.27 ± 13.31	
Females	197	2.64 ± 0.68		165	40.45 ± 11.51	
Age (years)			0.80 (0.495)			2.91 (0.035)
8–9	142	2.76 ± 0.70		127	45.15 ± 14.32	
10	100	2.68 ± 0.76		84	44.01 ± 12.36	
11	158	2.72 ± 0.67		117	40.59 ± 11.10	
12–13	49	2.59 ± 0.64		37	41.89 ± 11.61	

Notes: PAQ-C, Physical Activity Questionnaire for Older Children; MVPA, Moderate- to Vigorous- intensity PA; SD, Standard Deviation.

**Table 2:** Descriptive statistics, internal reliability coefficients and Pearson correlation coefficients (*n* = 449).

Variable (Range of Possible Score)	Mean (SD)	*α*	1	2	3	4	5	6	7	8	9
1. PA self-efficacy (1,4)	2.13 (0.59)	0.92									
2. PA preference (1,4) ^#^	2.86 (0.42)	0.85	0.55 ***								
3. Intrinsic motivation for PA (1,7)	5.34 (1.73)	0.86	0.52 ***	0.51 ***							
4. Identified regulation for PA (1,7)	5.03 (1.58)	0.81	0.45 ***	0.46 ***	0.70 ***						
5. Introjected regulation for PA (1,7) ^#^	3.05 (1.72)	0.72	0.52 ***	0.39 ***	0.47 **	0.49 ***					
6. External regulation for PA (1,7) ^#^	2.40 (1.35)	0.73	0.28 ***	0.14	0.10	0.21 **	0.53 **				
7. Autonomous motivation (1,7)	5.13 (1.52)	0.89	0.53 ***	0.53 **	0.88 ***	0.95 ***	0.50 **	0.19 **			
8. Controlled motivation (1,7) ^#^	2.64 (1.30)	0.80	0.45 ***	0.33 **	0.32 **	0.40 **	0.84 **	0.89 ***	0.40 ***		
9. PAQ-C score (1,5) ^¶^	2.71 (0.70)	0.78	0.63 ***	0.53 **	0.53 ***	0.42 ***	0.41 **	0.20 **	0.50 ***	0.34 **	
10. Objective MVPA (min/day) ^#,¶^	43.09 (12.74)	0.72	0.35 ***	0.24 *	0.29 **	0.22 **	0.24 **	0.13	0.31 **	0.22 **	0.39 **

Notes: PA, physical activity; PAQ-C, Physical Activity Questionnaire for Older Children; MVPA, Moderate- to vigorous- intensity PA. Partial correlation coefficients were reported with adjustment for gender and age. *α*, Cronbach’s alpha for variable 1–9, intraclass correlation coefficient across days for variable 10. ^¶^ The analyses between psychological correlates and PA behaviors were conducted among participants who provided valid accelerometer data (*n* = 365). ^#^ Significant differences between genders, *p* < 0.05 using independent *t*-tests. * *p* < 0.05, ** *p* < 0.01, *** *p* < 0.001.

**Table 3:** Hierarchical Multiple Regression Analyses Predicting the PAQ-C Score and Objectively Monitored MVPA (*n* = 365).

	PAQ-C Score					Accelerometry-Based MVPA				
	B	SE	*t*	*R*^2^	Δ *R*^2^	B	SE	*t*	*R*^2^	Δ *R*^2^
**Step 1**				0.01					0.04	
Age	0.01	0.03	0.26			−0.06	0.66	−1.06		
Gender	−0.07	0.07	−1.30			−0.16	1.38	−2.82 *		
BMI	−0.03	0.01	−0.64			−0.04	0.19	0.78		
Social desirability	0.05	0.01	0.91			0.04	0.06	0.68		
**Step 2**				0.46	0.45 **				0.17	0.13 **
Age	0.09	0.03	2.14 *			−0.02	0.63	−0.34		
Gender	−0.04	0.05	−0.96			−0.15	1.33	−2.72 *		
BMI	0.03	0.01	0.66			−0.07	0.18	1.37		
Social desirability	0.01	0.01	0.12			0.03	0.06	0.44		
PA self-efficacy	0.40	0.06	7.76 **			0.23	1.45	3.26 *		
PA preference	0.22	0.08	4.48 **			0.02	1.92	0.32		
Autonomous motivation	0.16	0.02	3.20 *			0.16	0.55	2.42 *		
Controlled motivation	0.05	0.02	1.19			0.03	0.62	0.48		

Note: B, standardized coefficients; SE, standard error; BMI, Body mass index; PA, physical activity; PAQ-C, Physical Activity Questionnaire for Older Children; MVPA, Moderate- to vigorous- intensity PA. * *p* < 0.05, ** *p* < 0.01.

## References

[1] Janssen I., LeBlanc A.G. (2010). Review systematic review of the health benefits of physical activity and fitness in school-aged children and youth. Int. J. Behav. Nutr. Phys. Act..

[2] World Health Orgnization (WHO) Global Recommendations on Physical Activity for Health. http://www.who.int/dietphysicalactivity/publications/9789241599979/en/.

[3] Kohl H.W., Craig C.L., Lambert E.V., Inoue S., Alkandari J.R., Leetongin G., Kahlmeier S. (2012). The pandemic of physical inactivity: Global action for public health. Lancet.

[4] Wang C., Chen P., Zhuang J. (2013). A national survey of physical activity and sedentary behavior of Chinese city children and youth using accelerometers. Res. Q. Exerc. Sport.

[5] Hong Kong Leisure and Cultural Services Department, China Health Exercise for All Campaign-Physical Fitness Test for Community: Final Summary Report. http://www.lcsd.gov.hk/healthy/physical_fitness/download/SummaryReport_en.pdf.

[6] Baranowski T., Anderson C., Carmack C. (1998). Mediating variable framework in physical activity interventions: How are we doing? How might we do better?. Am. J. Prev. Med..

[7] Sallis J.F., Prochaska J.J., Taylor W.C. (2000). A review of correlates of physical activity of children and adolescents. Med. Sci. Sports Exerc..

[8] Strauss R.S., Rodzilsky D., Burack G., Colin M. (2001). Psychosocial correlates of physical activity in healthy children. Arch. Pediatr. Adolesc. Med..

[9] Bandura A. (1986). Social Foundations of Thought and Action: A Social-Cognitive View.

[10] Norman P., Connor M. (2005). Predicting Health Behaviour: Research and Practice with Social Cognition Models.

[11] McAuley E., Blissmer B. (2000). Self-efficacy determinants and consequences of physical activity. Exerc. Sport Sci. Rev..

[12] Li M., Dibley M.J., Sibbritt D., Yan H. (2006). Factors associated with adolescents’ physical inactivity in Xi’an city, China. Med. Sci. Sports Exerc..

[13] Murnan J., Sharma M., Lin D. (2007). Predicting childhood obesity prevention behaviors using social cognitive theory: Children in China. Int. Q. Community Health Educ..

[14] Wu T.Y., Pender N. (2002). Determinants of physical activity among Taiwanese adolescents: An application of the health promotion model. Res. Nurs. Health.

[15] Wu T.Y., Pender N., Noureddine S. (2003). Gender differences in the psychosocial and cognitive correlates of physical activity among Taiwanese adolescents: A structural equation modeling approach. Int. J. Behav. Med..

[16] Huang W.Y., Wong S.H., Salmon J. (2013). Correlates of physical activity and screen-based behaviors in Chinese children. J. Sci. Med. Sport.

[17] Deci E.L., Ryan R.M. (1985). Intrinsic Motivation and Self-Determination in Human Behavior.

[18] Ryan R.M., Deci E.L. (2000). Self-determination theory and the facilitation of intrinsic motivation, social development, and well-being. Am. Psychol..

[19] Deci E.L., Ryan R.M. (2002). Handbook of Self-Determination Research.

[20] Ryan R.M., Deci E.L., Hagger M.S., Chatzisarantis N.L. (2007). Active human nature: Self-determination theory and the promotion and maintenance of sport, exercise, and health. Intrinsic Motivation and Self-Determination in Exercise and Sport.

[21] Owen K.B., Smith J., Lubans D.R., Ng J.Y., Lonsdale C. (2014). Self-determined motivation and physical activity in children and adolescents: A systematic review and meta-analysis. Prev. Med..

[22] Lonsdale C., Sabiston C.M., Raedeke T.D., Ha A.S., Sum R.K. (2009). Self-determined motivation and students’ physical activity during structured physical education lessons and free choice periods. Prev. Med..

[23] Wang C.J., Liu W., Sun Y., Lim B.C., Chatzisarantis N.L. (2010). Chinese students’ motivation in physical activity: Goal profile analysis using nicholl’s achievement goal theory. Int. J. Sport Exerc. Psychol..

[24] Pan C.Y., Tsai C.L., Chu C.H., Hsieh K.W. (2011). Physical activity and self-determined motivation of adolescents with and without autism spectrum disorders in inclusive physical education. Res. Autism Spect. Dis..

[25] Zhang J., Middlestadt S.E., Ji C.Y. (2007). Psychosocial factors underlying physical activity. Int. J. Behav. Nutr. Phys. Act..

[26] Epstein L.H. (1998). Integrating theoretical approaches to promote physical activity. Am. J. Prev. Med..

[27] Salmon J., Owen N., Crawford D., Bauman A., Sallis J.F. (2003). Physical activity and sedentary behavior: A population-based study of barriers, enjoyment, and preference. Health Psychol..

[28] Rodenburg G., Oenema A., Pasma M., Kremers S.P., Van de Mheen D. (2013). Clustering of food and activity preferences in primary school children. Appetite.

[29] Epstein L.H., Saelens B.E., Myers M.D., Vito D. (1997). Effects of decreasing sedentary behaviors on activity choice in obese children. Health Psychol..

[30] Trost S.G. (2007). State of the art reviews: Measurement of physical activity in children and adolescents. Am. J. Lifestyle Med..

[31] Adams S.A., Matthews C.E., Ebbeling C.B., Moore C.G., Cunningham J.E., Fulton J., Hebert J.R. (2005). The effect of social desirability and social approval on self-reports of physical activity. Am. J. Epidemiol..

[32] Census and Statistics Department Hong Kong 2011 Population Census—Summary Results. http://www.censtatd.gov.hk/hkstat/sub/sp170.jsp?productCode=B1120055.

[33] De Vries S.I., Bakker I., Hopman-Rock M., Hirasing R.A., Van Mechelen W. (2006). Clinimetric review of motion sensors in children and adolescents. J. Clin. Epidemiol..

[34] Esliger D.W., Copeland J.L., Barnes J.D., Tremblay M.S. (2005). Standardizing and optimizing the use of accelerometer data for free-living physical activity monitoring. J. Phys. Act. Health.

[35] Trost S.G., Loprinzi P.D., Moore R., Pfeiffer K.A. (2011). Comparison of accelerometer cut points for predicting activity intensity in youth. Med. Sci. Sports Exerc..

[36] Evenson K.R., Catellier D.J., Gill K., Ondrak K.S., McMurray R.G. (2008). Calibration of two objective measures of physical activity for children. J. Sports Sci..

[37] Wang J.J., Baranowski T., Lau W.C., Chen T.A., Pitkethly A.J. (2016). Validation of the physical activity questionnaire for older children (PAQ-C) among Chinese children. Biomed. Environ. Sci..

[38] Jago R., Baranowski T., Watson K., Bachman C., Baranowski J.C., Thompson D., Hernández A.E., Venditti E., Blackshear T., Moe E. (2009). Development of new physical activity and sedentary behavior change self-efficacy questionnaires using item response modeling. Int. J. Behav. Nutr. Phys. Act..

[39] Sallis J.F., Strikmiller P.K., Harsha D.W., Feldman H.A., Ehlinger S., Stone E.J., Williston J., Woods S. (1996). Validation of interviewer-and self-administered physical activity checklists for fifth grade students. Med. Sci. Sports Exerc..

[40] Deci E.L., Ryan R.M. Self-Determination Theory: An Approach to Human Motivaiton and Personality. http://www.psych.rochester.edu/SDT/questionnaires.php.

[41] Reynolds C.R., Paget K.D. (1983). National normative and reliability data for the revised children’s manifest anxiety scale. Sch. Psychol. Rev..

[42] Taylor R. (1990). Interpretation of the correlation coefficient: A basic review. J. Diagn. Med. Sonogr..

[43] Bandura A. (2004). Health promotion by social cognitive means. Health Educ. Behav..

[44] Cho M.H. (2004). The strength of motivation and physical activity level during leisure time among youth in South Korea. Youth Soc..

[45] Plotnikoff R.C., Costigan S.A., Karunamuni N., Lubans D.R. (2013). Social cognitive theories used to explain physical activity behavior in adolescents: A systematic review and meta-analysis. Prev. Med..

[46] Standage M., Sebire S.J., Loney T. (2008). Does exercise motivation predict engagement in objectively assessed bouts of moderate-intensity exercise? A self-determination theory perspective. J. Sport Exerc. Psychol..

[47] Thøgersen-Ntoumani C., Ntoumanis N. (2006). The role of self-determined motivation in the understanding of exercise-related behaviours, cognitions and physical self-evaluations. J. Sports Sci..

[48] Edmunds J., Ntoumanis N., Duda J.L. (2006). A test of self-determination theory in the exercise domain. J. Appl. Soc. Psychol..

[49] Fogelholm M., Kukkonen-Harjula K. (2000). Does physical activity prevent weight gain—A systematic review. Obes. Rev..

[50] Pelletier L.G., Fortier M.S., Vallerand R.J., Briere N.M. (2001). Associations among perceived autonomy support, forms of self-regulation, and persistence: A prospective study. Motiv. Emot..

[51] Silva M.N., Markland D., Carraca E.V., Vieira P.N., Coutinho S.R., Minderico C.S., Matos M.G., Sardinha L.B., Teixeira P.J. (2011). Exercise autonomous motivation predicts 3-yr weight loss in women. Med. Sci. Sports Exerc..

[52] Jeffery R.W., Epstein L.H., Wilson G.T., Drewnowski A., Stunkard A.J., Wing R.R. (2000). Long-term maintenance of weight loss: Current status. Health Psychol..

[53] Grieser M., Vu M.B., Bedimo-Rung A.L., Neumark-Sztainer D., Moody J., Young D.R., Moe S.G. (2006). Physical activity attitudes, preferences, and practices in African American, Hispanic, and Caucasian girls. Health Educ. Behav..

[54] Gao Y., Wang J.J., Lau P.W., Ransdell L. (2015). Pedometer-determined physical activity patterns in a segmented school day among Hong Kong primary school children. J. Exerc. Sci. Fit..

[55] Sallis J.F., Prochaska J.J., Taylor W.C., Hill J.O., Geraci J.C. (1999). Correlates of physical activity in a national sample of girls and boys in grades 4 through 12. Health Psychol..

[56] Brener N.D., Collins J.L., Kann L., Warren C.W., Williams B.I. (1995). Reliability of the youth risk behavior survey questionnaire. Am. J. Epidemiol..

[57] Prochaska J.J., Rodgers M.W., Sallis J.F. (2002). Association of parent and peer support with adolescent physical activity. Res. Q. Exerc. Sport.

[58] Dishman R., Darracott C.R., Lambert L.T. (1992). Failure to generalize determinants of self-reported physical activity to a motion sensor. Med. Sci. Sports Exerc..

[59] Podsakoff P.M., MacKenzie S.B., Podsakoff N.P. (2012). Sources of method bias in social science research and recommendations on how to control it. Annu. Rev. Psychol..

