# Correction: Wang, J.-J., et al. Psychological Correlates of Self-Reported and Objectively Measured Physical Activity among Chinese Children—Psychological Correlates of PA. *Int. J. Environ. Res. Public Health* 2016, *13*, 1006

**DOI:** 10.3390/ijerph13111124

**Published:** 2016-11-11

**Authors:** Jing-Jing Wang, Tom Baranowski, Patrick W. C. Lau, Tzu-An Chen, Shu-Ge Zhang

**Affiliations:** 1 Department of Physical Education, Faculty of Social Sciences, Hong Kong Baptist University, Hong Kong, China; wangjj@life.hkbu.edu.hk (J.-J.W.); sugar.zhangshuge@gmail.com (S.-G.Z.); 2 Children’s Nutrition Research Center, Department of Pediatrics, Balor College of Medicine, Houston, TX 77030, USA; Thomas.Baranowski@bcm.edu; 3 Center for Translational Injury Research, University of Texas Health Science Center at Houston, Houston, TX 77030, USA; Tzu-An.Chen@uth.tmc.edu

The authors wish to make the following correction to their paper [1]. In the Reference Section, References [32–40] should be renumbered [31] to [39]. Reference [31] should be renumbered as Reference 40. The correct references [31–40] should appear as follows:

31 Census and Statistics Department. *Population Census—Summary Results*; Census and Statistics Department: Hong Kong, China, 2011 Available online: http://www.censtatd.gov.hk/hkstat/sub/sp170.jsp?productCode=B1120055 (accessed on 25 April 2015).
32 De Vries, S.I.; Bakker, I.; Hopman-Rock, M.; Hirasing, R.A.; Van Mechelen, W. Clinimetric review of motion sensors in children and adolescents. *J. Clin. Epidemiol.* **2006**, *59*, 670–680.
33 Esliger, D.W.; Copeland, J.L.; Barnes, J.D.; Tremblay, M.S. Standardizing and optimizing the use of accelerometer data for free-living physical activity monitoring. *J. Phys. Act. Health* **2005**, *3*, 366–383.
34 Trost, S.G.; Loprinzi, P.D.; Moore, R.; Pfeiffer, K.A. Comparison of accelerometer cut points for predicting activity intensity in youth. *Med. Sci. Sports Exerc.* **2011**, *43*, 1360–1368.
35 Evenson, K.R.; Catellier, D.J.; Gill, K.; Ondrak, K.S.; McMurray, R.G. Calibration of two objective measures of physical activity for children. *J. Sports Sci.* **2008**, *26*, 1557–1565.
36 Wang, J.-J.; Baranowski, T.; Lau, W.C.; Chen, T.A.; Pitkethly, A.J. Validation of the physical activity questionnaire for older children (PAQ-C) among Chinese children. *Biomed. Environ. Sci.* **2016**, *29*, 177–186.
37 Jago, R.; Baranowski, T.; Watson, K.; Bachman, C.; Baranowski, J.C.; Thompson, D.; Hernández, A.E.; Venditti, E.; Blackshear, T.; Moe, E. Development of new physical activity and sedentary behavior change self-efficacy questionnaires using item response modeling. *Int. J. Behav. Nutr. Phys. Act.* **2009**, *6*, 20.
38 Sallis, J.F.; Strikmiller, P.K.; Harsha, D.W.; Feldman, H.A.; Ehlinger, S.; Stone, E.J.; Williston, J.; Woods, S. Validation of interviewer-and self-administered physical activity checklists for fifth grade students. *Med. Sci. Sports Exerc.* **1996**, *28*, 840–851.
39 Deci, E.L.; Ryan, R.M. Self-Determination Theory: An Approach to Human Motivation and Personality. Available online: http://www.psych.rochester.edu/SDT/questionnaires.php (accessed on 15 August 2014).
40 Adams, S.A.; Matthews, C.E.; Ebbeling, C.B.; Moore, C.G.; Cunningham, J.E.; Fulton, J.; Hebert, J.R. The effect of social desirability and social approval on self-reports of physical activity. *Am. J. Epidemiol.* **2005**, *161*, 389–398.
